# Internal Radiation Exposure Dose in Iwaki City, Fukushima Prefecture after the Accident at Fukushima Dai-ichi Nuclear Power Plant

**DOI:** 10.1371/journal.pone.0114407

**Published:** 2014-12-05

**Authors:** Makiko Orita, Naomi Hayashida, Hiroshi Nukui, Naoko Fukuda, Takashi Kudo, Naoki Matsuda, Yoshiko Fukushima, Noboru Takamura

**Affiliations:** 1 Department of Global Health, Medicine and Welfare, Nagasaki University Graduate School of Biomedical Sciences, Nagasaki, Japan; 2 Department of Radioisotope Medicine, Nagasaki University Graduate School of Biomedical Sciences, Nagasaki, Japan; 3 Department of Radiation Protection, Nagasaki University Graduate School of Biomedical Sciences, Nagasaki, Japan; 4 Department of Nursing, Hirosaki University Graduate School of Health Sciences, Hirosaki, Japan; University of California Davis, United States of America

## Abstract

As a result of the accident at the Fukushima Daiichi Nuclear Power Plant (FNPP) on 11 March 2011, a huge amount of radionuclides, including radiocesium, was released and spread over a wide area of eastern Japan. Although three years have passed since the accident, residents around the FNPP are anxious about internal radiation exposure due to radiocesium. In this study, we screened internal radiation exposure doses in Iwaki city of Fukushima prefecture, using a whole-body counter. The first screening was conducted from October 2012 to February 2013, and the second screening was conducted from May to November 2013. Study participants were employees of ALPINE and their families who underwent examination. A total of 2,839 participants (1,366 men and 1,473 women, 1–86 years old) underwent the first screening, and 2,092 (1,022 men and 1,070 women, 1–86 years old) underwent the second screening. The results showed that 99% of subjects registered below 300 Bq per body in the first screening, and all subjects registered below 300 Bq per body in the second screening. The committed effective dose ranged from 0.01–0.06 mSv in the first screening and 0.01–0.02 mSv in the second screening. Long-term follow-up studies are needed to avoid unnecessary chronic internal exposure and to reduce anxiety among the residents by communicating radiation health risks.

## Introduction

Due to the accident at the Fukushima Daiichi Nuclear Power Plant (FNPP) following the Great East Japan Earthquake on March 11, 2011, a huge amount of radionuclides including ^131^I (half-life: 8.0 d), ^134^Cs (2.1 y) and ^137^Cs (30 y) were released [1], resulting in the radioactive contamination of a wide area of eastern Japan [2]–[4].

Based on the experiences of the Chernobyl Nuclear Power Plant (CNPP) accident in 1986, it is known that internal radiation exposure due to ^131^I in the thyroid gland causes an increased risk of childhood thyroid cancer [5]. On March 17, 2011, the Director General of the Nuclear Emergency Response Headquarters of Japan initiated food control measures to minimize internal radiation exposure to ^131^I, and disposed of all contaminated raw milk.

A few months after the accident, the level of ^131^I decreased due to its short life, and thereafter ^134^Cs and ^137^Cs became the main radionuclides in the environment around FNPP. Since soil contamination with ^134^Cs and ^137^Cs may contaminate foods, such as mushrooms, fruits, and vegetables, internal radiation exposure to radiocesium causes some anxiety for residents around FNPP [6].

The accumulation of ^134^Cs and ^137^Cs in the human body is non-specific, except for weak accumulation in muscle and bone [5], [6], and its biological half-life in adults is about 70 days [7]. Internal radiation doses are determined using whole body counters (WBC), which are special instruments usually housed within or around nuclear facilities [8]. Several studies have assessed internal radiation exposure to radiocesium using WBC since the accident at CNPP [5], [9]. Hoshi et al. evaluated the ^137^Cs body burden in children residing in Bryansk Oblast, Russian Federation from 1991–1996 within the framework of the Chernobyl Sasakawa Medical Health and Medical Cooperation Project. They showed a relationship between ^137^Cs activity and the level of ^137^Cs soil contamination [10]. Contamination of soils by ^137^Cs continues even today, but the internal exposure dose in the population residing around CNPP is extremely limited [5], [9], [10], [11], [12].

**Figure 1:**
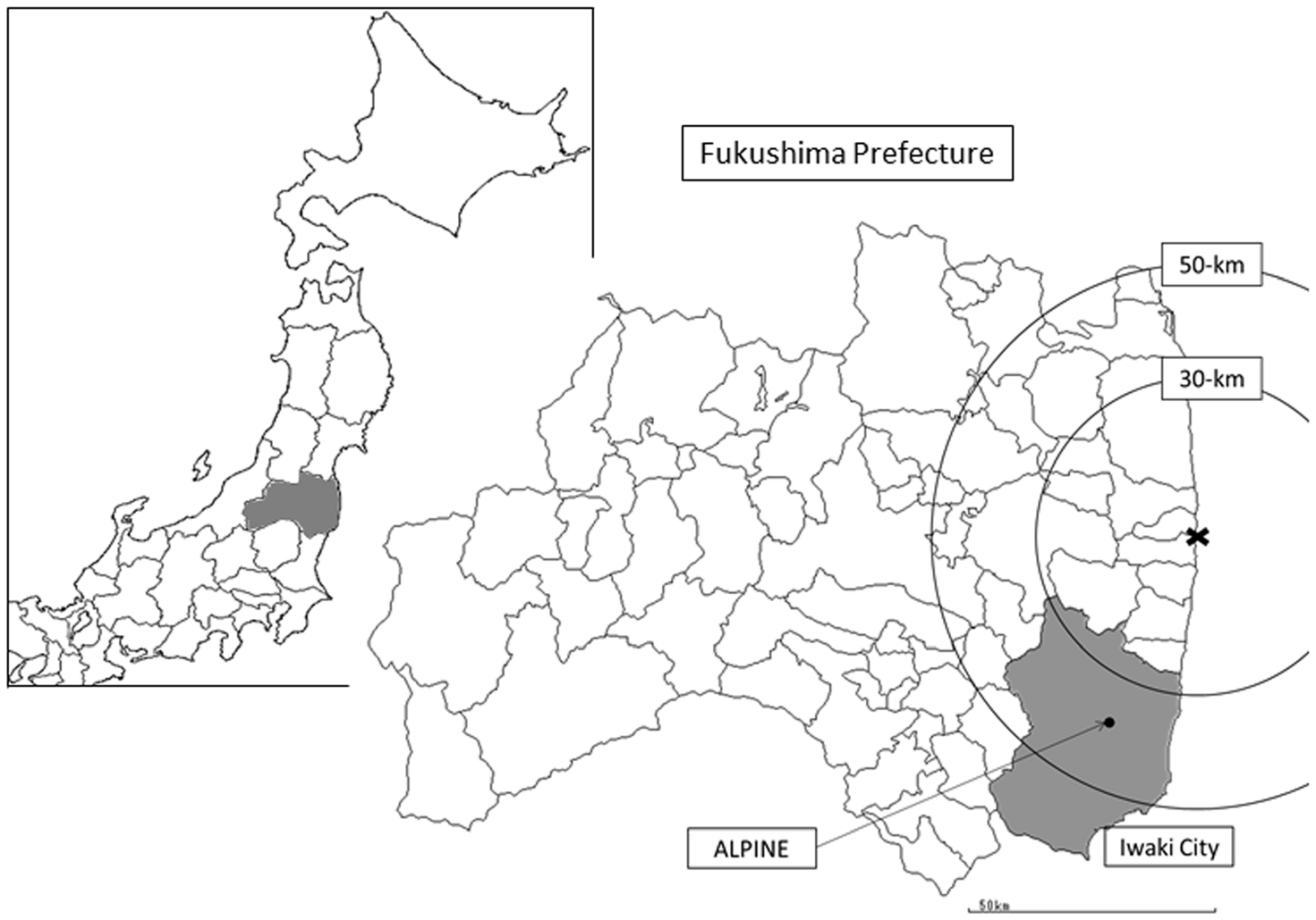
Location of Iwaki city in Fukushima Prefecture.

**Figure 2:**
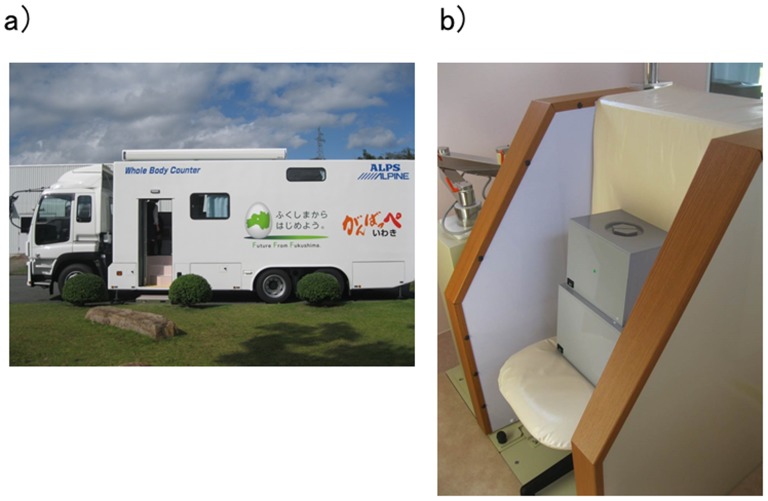
A vehicle-mounted type of WBC introduced in ALPINE, Iwaki city, Fukushima Prefecture. a) The large-size car mounted type of WBC. b) The chair equipped with a NaI (T1) scintillation detector.

Iwaki city is located to the southeast of the Fukushima prefecture (Figure 1). It is one of the largest cities in Japan in terms of land area, and the second largest city in Fukushima prefecture in terms of population. The city is located within a 30–60 km radius around FNPP but was not designated as an evacuation zone. Nevertheless, many residents voluntarily evacuated from Iwaki city due to their anxiety about radiation exposure [13]. At ALPINE electronics, Inc. (ALPINE), which has its head office in Iwaki city, some workers left the company due to anxiety about the possible health effects on their family and themselves. Workers who remained at the company felt anxiety about potential health effects due to radiation exposure. This situation led ALPINE to conduct WBC (Figure 2) to assess the internal radiation exposure of their employees in order to minimize the anxiety of their workers and their families. In this study, we reported the results of the internal body burden screening conducted at ALPINE from October 2012 to February 2013 (first screening) and from May to November 2013 (second screening), and we evaluated internal radiation exposure to radiocesium in residents living in Iwaki city.

## Material and Methods

This study was approved by the ethics committees of Nagasaki University Graduate School of Biomedical Sciences (No. 13013171). Written informed consent was obtained from all study participants and/or their parent in case of children. The study was conducted at ALPINE in Iwaki city (Fukushima Prefecture), which is located in the 30–60 km radius from FNPP. The first screening was conducted from October 2012 to February 2013, and the second screening was conducted from May to November 2013. The interval between the first and second screening was at least 6 months to allow comparison of the change in internal radiation doses between the two periods. Study participants were employees of ALPINE and their family members living in Iwaki city at the time of the accident at FNPP. The study participants (shown in Table 1) included 2,839 participants (1,366 men and 1,473 women, 1–86 years old) who underwent the first screening and 2,092 (1,022 men and 1,070 women, 1–86 years) who underwent the second screening. Among them, 1,562 participants underwent both screenings, 1,277 underwent the first screening only, and 528 underwent the second screening only.

**Table 1:** Number of the study participants.

	First screening (2012)		Second screening (2013)	
Age	Total	Sex (men/women)	Total	Sex (men/women)
0–9	181	92/89	122	66/56
10–19	203	93/110	125	53/72
20–29	341	144/197	248	117/131
30–39	666	340/326	499	268/231
40–49	852	399/453	611	282/329
50–59	435	218/217	365	182/183
60–69	109	55/54	81	36/45
70–	52	25/27	41	18/23
Total	2839	1366/1473	2092	1022/1070

A gamma-spectrometer (NLW-22331, Fuji Electric Co., Ltd., Japan) was used to measure the radiocesium body burden. The counter was equipped with a 3×5×16 inch NaI (Tl) scintillation detector with adjustable height and angle, which was designed to face the back of the examinee sitting in a chair. The gamma-ray energy spectrum was obtained using a multi-channel analyzer equipped with preinstalled gamma analysis and calibration software. The minimum detectable radioactivity per body for ^134^Cs and ^137^Cs was 200 Bq and 220 Bq, respectively. The overall system was configured and assembled by Fuji Electric (Tokyo, Japan). Internal radiation exposure was measured as both total body exposure and concentration by body weight (Bq/kg). We also used WBC to measure radioactive potassium (^40^K) levels; these had a constant level of 0.0117% of the total body potassium [8].

The conversion from the measured body burden to the committed effective dose throughout the life by internal exposure was calculated using MONDAL 3 (monitoring to dose calculation ver. 3) software developed by the National Institute of Radiological Science (Chiba, Japan) [14]–[17]. This program has been validated at the National Institute of Radiological Science and contains preinstalled information regarding the essential parameters reported in the International Committee on Radiation Protection (ICRP) publications 30, 56, 66, 67, 69, and 71. Intake of ^134^Cs and ^137^Cs were assumed to occur from chronic consumption of food containing ^134^Cs and ^137^Cs since 12 March, 2011.

Data are expressed as median (minimum to maximum) and mean ± standard deviation. Statistical analysis was performed using SPSS statistics 18.0 (IBM Japan, Tokyo, Japan).

## Results

Table 2 described number of participants with detected radioactivity, the detection rate and the internal radioactivity of ^134^Cs and ^137^Cs during the screening. Mean values of ^40^K in the first and second screening were 3,681±1,259 Bq per body and 3,366±1,349 Bq per body, respectively. Maximum values in the first and second screening were 8,884 Bq and 9,628 Bq per body, respectively.

**Table 2:** Detection rate and internal radioactivity.

		Number of participants with detected radioactivity (Detection rate, %)		Internal radioactivity (Bq/Body) Median (minimum–maximum)	
Screening	n	^134^Cs	^137^Cs	^134^Cs	^137^Cs
First	2839	12 (0.42%)	37 (1.30%)	222 (200–495)	258 (221–565)
Second	2092	0 (0%)	12 (0.57%)	<200*	246 (225–289)

^*^: <200 as below detection limit, no range can be given.

In the first screening, radiocesium (either ^134^Cs and ^137^Cs or both) was detected in 44 (1.55%) of the 2,839 subjects. ^134^Cs was detected in 12 subjects (0.42%) and ^137^Cs was detected in 37 subjects (1.30%). The median value of ^134^Cs per body was 222 Bq, ranging from 200–495 Bq, with a mean value of 280±96 Bq. The median value of ^137^Cs per body was 258 Bq, ranging from 221–565 Bq, with a mean value of 285±75 Bq. Both ^134^Cs and ^137^Cs were detected in five subjects (0.18%). The total amount of ^134^Cs and ^137^Cs ranged from 200–822 Bq/body, the median value was 352 Bq/body, the mean value was 366±154 Bq/body, and the concentration was 2.3–15.0 Bq/kg. Short-lived radionuclides such as ^131^I were not detected in any subjects.

In the second screening, ^137^Cs was detected in 12 (0.66%) of the 2,090 subjects, and ^134^Cs was not detected in any study participants. The median value of ^137^Cs per body was 246 Bq, ranging from 225–289 Bq/body, the mean value was 248±18 Bq, with a concentration of 2.8–5.5 Bq/kg.

**Figure 3:**
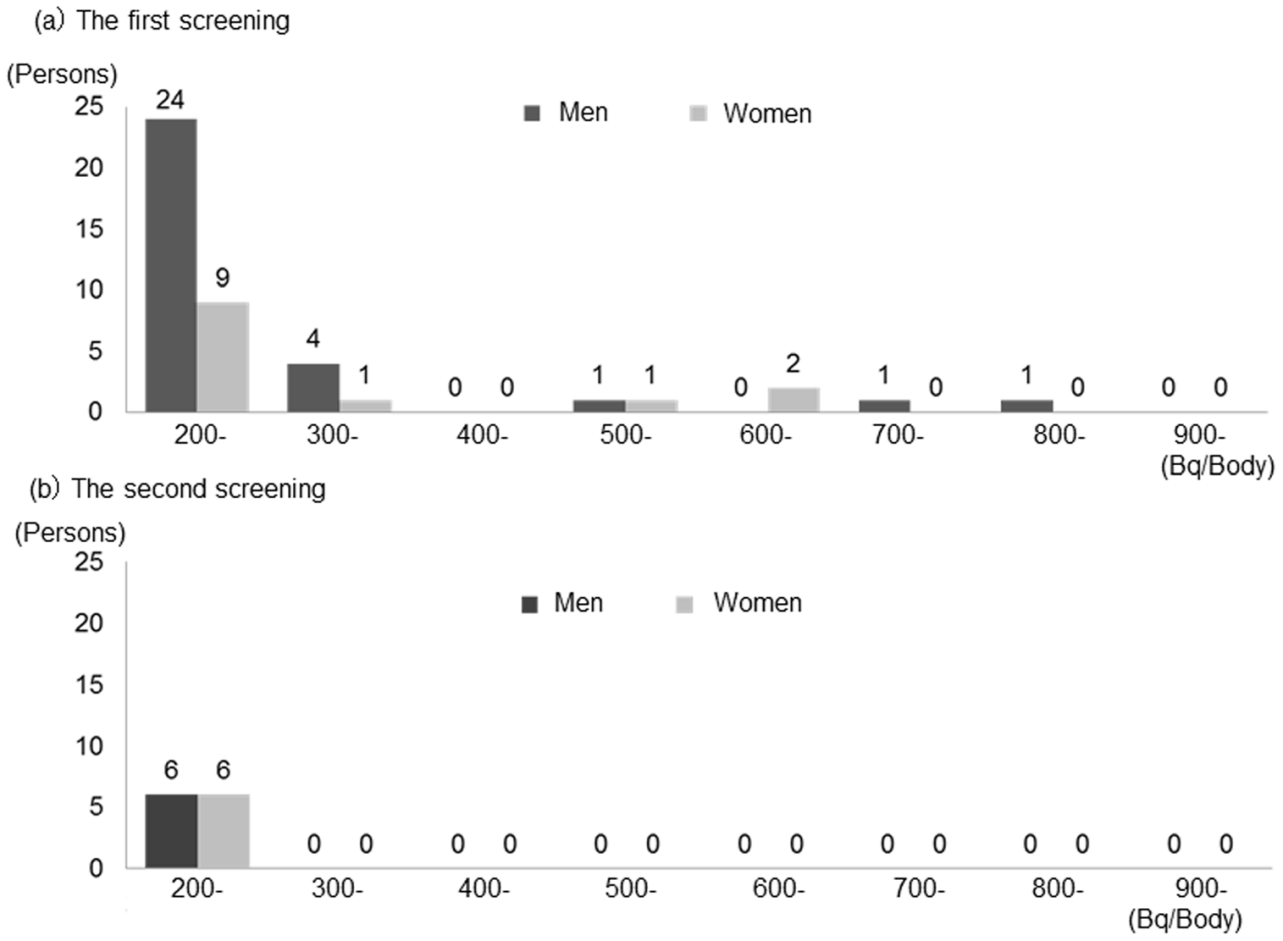
Participants detected with radiocesium in the first screening (a) and the second screening (b).

The radiocesium body burden was below 300 Bq per body for 99% of all subjects in the first screening, and all subjects had levels below 300 Bq per body in the second screening (Figure 3). No major gender difference was observed in either screening. Among the 44 subjects in whom radiocesium was detected in the first screening, 26 subjects underwent the second screening; radiocesium was detected in none in the second screening.

**Figure 4:**
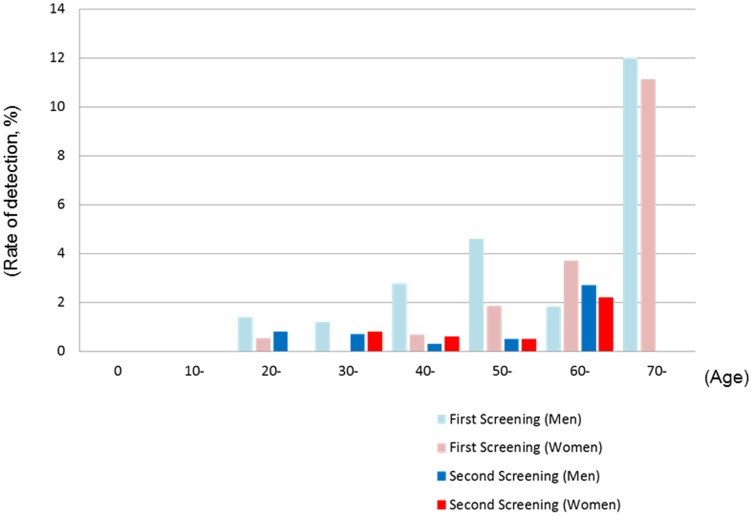
Detection rate of radiocesium by age group.

Figure 4 shows the detection rate of radiocesium by age group. In younger subjects (less than 20 years old), none showed radiocesium in either screening. Although the detection rate of radiocesium was slightly higher in older subjects (more than 70 years old) in the first screening, no similar tendency was observed in the second screening.

Calculated internal exposure doses based on the committed effective dose are shown in Table 3. The committed effective dose ranged from 0.01–0.06 mSv in the first screening and 0.01–0.02 mSv in the second screening. No value exceeded 1 mSv.

**Table 3:** Number of participants with committed effective doses (mSv).

Screening	n	<0.01	0.01 -	0.02 -	0.03 -	0.04 -	0.05 -	0.06 -	0.07<
First	44	-	31 (70.5%)	7 (15.9%)	1 (2.3%)	3 (6.8%)	1 (2.3%)	1 (2.3%)	0(0%)
Second	12	-	2 (16.7%)	10 (83.3%)	0(0%)	0(0%)	0(0%)	0(0%)	0(0%)

## Discussion

In this study, we examined ^134^Cs and ^137^Cs body burden using WBC among residents in Iwaki city, Fukushima Prefecture, and we showed that 99% of subjects were below 300 Bq per body in the first screening, and all subjects were below 300 Bq per body in the second screening. Several studies have been conducted to assess internal radiation exposure in residents since the accident at FNPP. Harada et al. evaluated dietary exposure to ^134^Cs and ^137^Cs by assessing duplicate food samples in three areas of Fukushima prefecture (Kawauchi village, Soma city, and Minamisoma city, which are all within a 20–50 km radius of the FNPP) in August and September 2012 [18]. They found median values of <0.40 to 0.54 Bq/day for ^134^Cs and 0.52 to 0.86 Bq/day for ^137^Cs in duplicate food samples. The maximum estimated annual dose was 120 µSv/y in the Soma area. These radiation levels were much lower than the external radiation dose rates. Tsubokura et al. also assessed the levels of internal radiocesium exposure in Kawauchi village from April 2012 to March 2013 [19]. Five of the 149 residents examined had detectable levels of radiocesium exposure, the median detected level was 333 Bq/body, and 5.3 Bq/kg. Our present results also suggest that levels of internal radiation exposure in Iwaki city are extremely low, and that countermeasures to minimize internal radiation exposure were effective in Fukushima. The Ministry of Health, Labor, and Welfare of Japan initiated food monitoring on March 16, just after the accident, and set provisional regulation values for contaminated food and water on March 17 [12]. On March 21, the government announced a ban on sales of raw milk shipped from Fukushima and neighboring prefectures. These food sources were screened, and those exceeding provisional standard values for radioiodine and radiocesium were eliminated from the market [12].

In our present study, radiocesium was detected in 44 subjects in the first screening. Of these, 26 subjects underwent a second screening and radiocesium was detected in none. Tsubokura et al. evaluated the ^137^Cs body burden in 2,831 children using WBC from September 2011 to September 2012 in Minamisoma City, which is located 23 km north of the FNPP [20]. They calculated monthly percentages of subjects with positive cesium exposure and reported a clear declining trend from September 2011 (57.5%) to September 2012 (0%), which remained at zero after June 2012 [20]. In our study, we assumed that the level of radiocesium detected in the first screening was close to the detection limit and was below the detection limit in the second screening due to the biological half-life and limited intake of radiocesium.

Our study also showed that in younger subjects (less than 20 years old), radiocesium were detected in none in either screening. Minamisoma municipal office conducted the screening of internal doses in 3,390 children, including primary and junior high school students, using WBC from April to September 2013, and it reported on their website (in Japanese) that radiocesium was only detected in one child (7.2 Bk/kg) [21]. These results suggest that the levels of internal radiation exposure in children are extremely low.

Generational, regional, and seasonal differences might influence dietary habits and preferences, and this may have affected the internal radiocesium body burden [22], [23]. In our study, the detection rate of radiocesium was slightly higher in older subjects (more than 70 years old) in the first screening, but this tendency was not observed in the second screening. The differences between the two screenings are probably due to the substantial decrease in numbers of persons in whom radiocesium was detected in the second screening. Recently, we calculated external and internal exposure due to artificial radionuclides by measuring the soil and local agricultural products in Kawauchi village, Fukushima Prefecture, using gamma spectrometry, and we reported that dietary differences between generations might contribute to the committed effective doses [6]. Therefore, during our second screening, we issued a questionnaire survey to subjects about their dietary habits, including the ingestion of edible wild plants, mushrooms, and fruits, but we found no differences in dietary habits between subjects in whom we detected radiocesium and in subjects we did not (data not shown). This may be because the shipment and ingestion of contaminated food were restricted, and ingestion of wild foods containing radiocesium decreased following the dissemination of safety information. Although the potential risk may be extremely low in Fukushima Prefecture now, identification of risk factors for internal exposure to radiocesium should be continuously conducted.

Our results show that internal exposure doses were also very low in residents living in Iwaki city. Fukushima prefecture has published the results of WBC screenings conducted by each local authority and by Fukushima prefecture on their website (in Japanese) [24], [25]. The total number of subjects was 178,630, including residents in Fukushima Prefecture and evacuees in Niigata Prefecture. Of these, 178,604 (99.9%) showed values of a committed effective dose of <1 mSv; the maximum was 3 mSv in two people [25]. Tsubokura et al. also evaluated the radiocesium body burden of 1,432 children and 8,066 adults using WBC at Minamisoma City in September 26, 2011, to March 31, 2011, and they reported that the committed effective doses were less than 1 mSv in all but one subjects [26].

There are several limitations to this study. We conducted our study only in Iwaki city, and included only the employees of ALPINE and their families. Since these persons live in urban areas of Iwaki city, they might have had fewer opportunities to consume locally produced vegetables, edible wild plants, and mushrooms, which might have led to sampling bias. A possibility also exists that the occupations are biased in the present study because the socio/economic backgrounds of the study participants could not be evaluated in this study. Further comprehensive approaches, including the evaluation of external doses, are needed.

In conclusion, we evaluated internal radiation exposure in Iwaki city, Fukushima Prefecture, using WBC, and we confirmed that the committed effective doses in residents are sufficiently low and comparable to the public dose limit (<1 mSv). However, while we continue to communicate the radiation health risks with residents, follow-up studies are needed to avoid unnecessary chronic internal exposure and to reduce anxiety among the residents.
